# Improving Financial Management via Contemplation: Novel Interventions and Findings in Laboratory and Applied Settings

**DOI:** 10.3389/fpsyg.2017.00327

**Published:** 2017-03-07

**Authors:** Ben Harkin

**Affiliations:** Department of Psychology, University of Sheffield, Western BankSheffield, UK

**Keywords:** contemplation, avoidance, debt, financial mismanagement, estimates of expenditure, credit union loan applicants

## Abstract

The present research tackles two main areas of financial mismanagement, namely avoiding debt-related information and underestimating expenditure. We draw upon research which has shown that inviting people to think about reasons for avoiding something actually serves to reduce the likelihood that they will then avoid it, and potentially improves what they know about it. Therefore, in three studies we investigated if prompting participants to contemplate their debt (Studies 1 and 2) and expenditure (Study 3) would decrease avoidance of debt-related information and improve estimates of expenditure, respectively. Conform to our expectations prompting contemplation via questionnaire (Study 1) and video (Study 2) reduced avoidance of debt-related information. In other words, contemplation reduced the likelihood that people would avoid viewing their risk of debt. The success of prompting contemplation via video offers a new and important addition to the literature on contemplation, which has previously focused on using the traditional questionnaire format. In Study 3 we observed that contemplation improved the estimates of expenditure that loan applicants at a credit union provided. Specifically, contemplation resulted in participants providing larger and more detailed accounts of their expenditure, and increased the agreement between staff and clients for the number of expenditure items provided by the clients. In sum, these findings suggest that contemplation in the context of the above financial decision-making is a robust intervention, as it was effective for different types of interventions (questionnaire and video), behaviors (avoidance of debt-related information and improving estimates of expenditure), and samples (students and university staff; Studies 1 and 2 *and* loan applicants at a credit union; Study 3). We discuss the theoretical, policy and applied impact of these findings, and highlight limitations and considerations for future research.

## Introduction

Despite good financial management being an important aspect of an individual's success (Jorgensen, 2007), it is an alarming observation that people are generally poor at managing their finances (see Shapiro and Burchell, 2012; Ceschi et al., 2014). Explanations for financial mismanagement have been attributed to an array of factors, such as financial locus of control (Livingstone and Lunt, 1992), conscientiousness (Bernerth et al., 2012), debt-related attitudes (Webley and Nyhus, 2001), optimistic financial expectations (Brown et al., 2005), and demographics (Lea et al., 1993). Another factor, and the focus of the present paper, is the role of avoidance in financial mismanagement. The severity and prevalence with which people avoided their finances lead Burchell (2003) to call it a *phobia*, i.e., a psychosocial syndrome defined by avoidant attitudes and behaviors with respect to dealing with personal finances (Shapiro and Burchell, 2012). Conform to this the National Savings and Investment Survey (2012) revealed that, of the Britons who worry about their finances daily, 90% do not monitor their finances at least once a month. Furthermore, 83% of people do not use income/expenditure diaries, and 84% do not withdraw a set weekly amount that they can spend (National Savings and Investment Survey, 2012). Narayan et al. (2011) also identified finances as a common area that people avoided in their day to day life, and that this occurred independently of demographic factors. Avoidance such as this is further complicated as debt is one of the most taboo topics to discuss between couples, close friends and with parents. As observed in data which highlighted that 41% of the UK population identify finances as their biggest cause of stress, yet 46% say finances are a private matter not open to discussion (Legal and General, 2014). More seriously, it has been observed that between 75 and 83% of those with serious debt problems do not seek advice on how to deal their debt (Hatcher, 1994; Money Advice Service, 2013).

So we have seen that avoidance is a common, pathological and detrimental approach that people adopt in relation to their finances and debt. Indeed, a likely outcome is that it reduces the accuracy of the financial information that people provide (McCloud et al., 2013). As Shapiro and Burchell (2012) reported, people who avoid their finances know less about their own financial details, e.g., monthly expenditure, amount in bank account. A specific example of this was observed in a 2016 survey which reported that people underestimated their yearly household expenditure by £1,459, estimating it at £2,528 compared to the £3,987 they actually spent, a 37% discrepancy which equates to a deficit of £122 per month (Santander, 2016). We propose that avoidance underlies this, as people likely have access to all the financial information they need, yet their avoidance of it results in them being unable and/or unwilling to appropriately use it (see definition of avoidance below; McCloud et al., 2013). Yakoboski and Dickemper (1997) observed a more specific relationship between fear, avoidance and lack of knowledge, as they reported in their analyses of the 1997 Retirement Confidence survey that 74% of respondents did not know how much they needed to budget for their retirement (see also Bernheim et al., 2000). Interestingly of those who claimed to know this figure (e.g., expenditure to income ratio), most could not give it when asked. When probed, a primary reason given for not knowing how much they needed to save was that they were afraid of finding out the answer (see “The Ostrich Problem” proposed by Webb et al., 2013). In this case, the fear of not having saved enough results in them avoiding calculating how much they have actually or need to save. Worryingly people adopt the same taboo and avoidant perspective of their expenditure/budgeting as they do with their debt, i.e., 36% will not discuss their expenditure with their partner, and 25% leave their financial planning to chance (Legal and General, 2014). Unsurprisingly, avoidant attitudes to financial planning come with negative financial outcomes: Those who avoided dealing with their finances—e.g., do not track their expenditure—saved half the amount compared to those who did not avoid their finances (£53.47 vs. £104.39, respectively; National Savings and Investment Survey, 2012). As avoiding finances is a prevalent phenomenon with negative financial outcomes, it is important to understand what motivates avoidance of financial information.

### Why do people avoid financial information?

McCloud et al. (2013) defined “information avoiders” as “those who take steps to actively and purposefully *avoid learning* about or *being exposed* to information [emphasis added]” (p. 1950). This poses the question as to what type of information are people likely to avoid? Sweeny et al. (2010) proposed that people avoid information that: (i) threatens a strong personal belief (e.g., of being healthy and not likely to get cancer; Hart et al., 2009), (ii) requires undesired behavior (e.g., having to undergo a mastectomy if a lump in breast is malignant; Ajekigbe, 1991), and/or (iii) produces negative emotions (e.g., avoiding HIV results due to fear of negative psychological impact; Lyter et al., 1987). Thus, financial information and particularly negative financial information (i.e., debt, spending more than you save) taps into the three components that Sweeny et al. (2010) identified as necessary to motivate avoidance. For example, being in debt and/or receiving information that you are in debt likely: (i) threatens the *belief* that one is financially responsible, (ii) requires *undesired action* of seeking advice from a Citizens Advice Debt Unit, and (iii) causes *embarrassment* if others were to find out about their situation. More concretely, Narayan et al. (2011) proposed that financial information evokes a form of “active information avoidance,” which serves as a form of self-preservation to save the person from distress in the short-term. This may help explain the negative feedback loop that can occur between avoidance and an increasingly poorer financial situation. This fits with what Carver (2006) defined as “anti-goals,” where a person who does not want to accept that this is them, increases the distance between the present situation (poor financial situation) and the anti-goal (not wanting this to be the case), which results in avoidance. So while avoidance reduces stress in the short-term, it actually serves to make the original financial situation worse (i.e., increasing interest and/or court fines) and escalates the threats that they receive (i.e., more warning of court action letters, bailiffs at their home). Interestingly people who suffer from anxiety disorders adopt similar strategies. In that, to decrease their anxiety they actively avoid those stimuli which made them feel anxious, however, this paradoxically only serves to maintain their anxious responding to such stimuli, as it impedes new learning, and does not allow them to challenge false beliefs (Salters-Pedneault et al., 2004). Together this explains why avoiding finances is an entrenched behavior that is difficult to overcome without intervention.

### An intervention that reduces avoidance

Interventions based on “contemplation” have proven effective in reducing avoidance of threatening information (see Howell and Shepperd, 2013). In Prochaska and DiClemente's (2005) transtheoretical model of behavior change, they identified contemplation (i.e., considering the pros and cons of ones behaviors) as a necessary part of positive behavior change. In the context of information avoidance, Howell and Shepperd (2013) defined contemplation as thinking about why an individual would and would not avoid information. In one study they presented participants with questions inviting them to contemplate, their risk of diabetes, such as: “It would be useful to know my risk for diabetes” and “Learning that I am at high risk for diabetes would require me to take action I would rather not take”. Participants then chose to view their risk of diabetes. In accord with their hypotheses, fewer participants in the contemplation condition avoided the information about their risk for diabetes (25%) compared to those in the control condition (40%). Thus, asking participants to contemplate how they would deal with finding out their risk for diabetes decreased the likelihood that they would avoid viewing their risk of diabetes. As a result, the present paper used contemplation in the context of financial information, where the aim was to decrease avoidance of debt-related information and improve the estimates of expenditure that people provided. The need for effective interventions is also underlined by a body of research that indicates a strong relationship between the fear of economic crisis/being in debt and poor health outcomes. For example, Giorgi et al. (2015) showed that social support and job stress mediated the relationship between fear of economic crisis and health, a finding that was supported in a recent review of the literature by Mucci et al. (2016). In addition, research has indicated that difficulties in repaying debt is independently associated with suicidal ideation (see Hintikka et al., 1998), with those in debt being twice as likely to think about suicide compared to those not in debt (see Meltzer et al., 2011). As discussed previously, fear of an outcome (i.e., general economic crisis/personal debt) and/or suicidal thinking may paradoxically serve to encourage poorer financial mismanagement. Whereby, a person adopts a “head in the sand” approach as they feel overwhelmed by their present financial situation (real or imagined) and so choose to avoid dealing with it head on. Thus, considering the present economic climate and the stressors that this brings to an individual, there is a definitive need for interventions which encourage people to not avoid their debts and increase what they know about their own financial situation.

### Rationale for the present studies and hypotheses

To reiterate, we have seen that avoiding debt and finances is a prevalent phenomenon, one that likely reduces the accuracy of the financial information that people give. Fortuitously, contemplation is effective in reducing avoidance of information which people experience as threatening, and so in three studies we use contemplation to decrease avoidance of debt-related information and improve estimates of expenditure. As such, we address a key point highlighted by the finance researcher Winerman (2004), who stated that although “researchers have examined a multitude of possible psychological factors” they “have reached few conclusions” and that “at present … I don't think we have the research base to know exactly what is needed” to reduce problem debt (p. 62). With this in mind we now highlight the rationale for each study, and state the hypothesis in each case.

*Study 1: Reducing avoidance of debt-related information*. In Study 1 we modified Howell and Shepperd's (2013) questionnaire which was originally designed to prompt contemplation of diabetes and applied it to debt-related issues, this was then followed by the choice to view (or not) their risk for debt (debt avoidance task). This allowed us to answer a key empirical question identified by Howell and Shepperd (2013): “we do not know whether contemplation can reduce information avoidance in non-health domains” (p. 1701). Thus, our first *general* hypothesis was:

*H1*: Prompting participants to contemplate the cons of avoiding and pros of not avoiding debt-related information would decrease the likelihood that they would avoid debt-related information.

*Study 2: Using a video to prompt contemplation of debt-related avoidance*. Previous interventions have prompted contemplation via the traditional questionnaire method (Howell and Shepperd, 2013; Study 1 of this paper). In contrast, in Study 2 we prompted contemplation of debt-related issues by asking participants to watch a video. This video presented an expert in financial advice discussing why avoiding debt makes that situation worse (i.e., prompting contemplation the cons of avoiding debt) and that solutions/advice are available (i.e., prompting contemplation of the pros of getting help). As such, Study 2 quantifies the effect of watching a video on debt-related issues on the likelihood of viewing (or not) their risk of debt. This lead to our second *task-specific* hypothesis:

*H2:* Prompting participants to contemplate the cons of avoiding and pros of not avoiding debt-related information *via video* would decrease the likelihood that they would avoid debt-related information.

*Study 3: Improving estimates of expenditure in an applied setting*. Studies 1 and 2 investigated if prompting participants to contemplate debt-related issues would *decrease* the likelihood that they would avoid viewing their risk of debt. In Study 3 we now investigated a different proposition: Would contemplation *improve* the financial information that credit union loan applicants provided? Our rationale for focusing on this financial behavior in this group was four-fold. Firstly, people generally (see above; Santander, 2016) and this group specifically (as identified by staff at the credit union) have a tendency to underestimate their expenditure. Secondly, contemplation potentially encourages a degree of self-reflection, a process associated with greater self-control and self-regulation (Howell and Shepperd, 2013). This suggests that after contemplation, decision-making will be more thorough, detailed and personally beneficial (Yeung and Summerfield, 2012). In other words, contemplating expenditure may encourage people to give more accurate estimates of expenditure. Thirdly, staff used expenditure to help decide if the client could afford the loan they were applying for. Thus, a more accurate estimate of expenditure would benefit the staff in terms of the expediency of the loan application process. Finally, we propose that using such a sample was a more vigorous test of contemplation than occurred in our earlier studies. In that, the people who went to this credit union likely had more complex financial and social histories than the students and university staff who participated in Studies 1 and 2. For example, ~87% of credit loan applicants were in receipt of child benefit, 58% were not employed (vs. 5.1% National Average; Office for National Statistics, 2016), 63% had been in receipt of a social fund loan, and 67% have used high cost lenders (see Table 1). Unfortunately, an analysis of peoples financial histories and behaviors has identified a relationship between these demographic factors and poor financial management (i.e., ineffective planning for financial event, and having less self-efficacy and confidence in financial management; Money Advice Service, 2015), with financial illiteracy further related to poor financial outcomes (Hastings and Tejeda-Ashton, 2008). Thus, an intervention that improves financial estimations will be of benefit to credit union loan applicants and staff alike. With these points in mind we proposed the following *applied* hypothesis:

**Table 1:** **Demographic characteristics of CNEDCU clients**.

	***N***	**%**
***N* =**	**81**	**100**
**GENDER**		
Female	71	87.7
Male	10	12.3
Age		
<30	28	34.6
30–34	15	18.5
35–39	12	14.8
40–44	7	8.6
45–49	9	11.1
50–54	2	2.5
55–59	3	3.7
60–64	0	0.0
65–69	0	0.0
>70	1	1.2
Not Specified	4	4.9
Mean (SD)	34.36 (10.50)	
**NUMBER OF CHILDREN**		
0	6	7.4
1	33	40.7
2	20	24.7
3	14	17.3
4	5	6.2
5	1	1.2
6	1	1.2
Not Specified	1	1.2
**HOUSING TYPE**		
Own home	5	6.2
Rent	69	85.2
Living with parents	6	7.4
Other	1	1.2
**LANDLORD**		
The council	39	48.1
Housing association	15	18.5
Private	21	25.9
Not specified	6	7.4
**SERVED HMO**		
Yes	1	1.2
No	80	98.8
**EMPLOYMENT STATUS**		
Full time	13	16.0
Part Time	20	24.7
Unemployed	34	42.0
Retired	1	1.2
Home-maker	12	14.8
Not specified	1	1.2
**COUNTRY OF BIRTH**		
UK	79	97.5
Not specified	2	2.5
**COUNTY COURT JUDGMENTS**		
Yes	12	14.8
No	69	85.2
**DECLARED BANKRUPTCY**		
Yes	2	2.5
No	79	97.5
**USED HIGH COST LENDERS**		
Yes	54	66.7
No	27	33.3
**USED SOCIAL FUND**		
Yes	51	63.0
No	29	36.3
Not specified	1	1.2

*H3:* Prompting credit union loan applicants to contemplate their expenditure would improve their estimates of expenditure. We expected that this improvement would occur in three areas: (i) *Thoroughness*: more expenditure information would be provided; (ii) *Totals*: larger estimates of expenditure, i.e., clients give an estimate of expenditure that more closely matches what they actually spend; (iii) *Discrepancies between clients and staff* : greater agreement between clients and staff for the above two measures.

## Study 1: reducing avoidance of debt-related information

### Participants

*N* = 40 participants at a large university in the UK provided written informed consents. The average age was 19.25 (*SD* = 1.15) and there were 27 females and 13 males. Participants received course credit for taking part. Participants completed a consent form, and were debriefed at the end of the study. British Psychological Society ethical requirements were met.

### Apparatus

A computer presented questionnaire items specific to each condition (contemplation or control) to participants. The Psychopy open source software package was used to design and present these items.

### Design

A single factor, with two levels (contemplation vs. control) design was employed to compare the effect of contemplating debt-related issues vs. not contemplating on avoidance of debt-related information. Participants were randomly assigned to either the debt contemplation (experimental) or no contemplation (control) condition. The dependent variable was the choice between viewing (non-avoidance) and not viewing (avoidance) their risk of debt in the future.

### Procedure

Participants sat in front of a computer screen. The first screen provided instructions on how to complete the questionnaire. Participants in the debt contemplation condition completed 18 questions on the cons of avoiding and pros of not avoiding debt-related information. We modified these questions from those originally used by Howell and Shepperd (2013) who asked participants to contemplate their risk of diabetes. For example, we modified the question “Learning that I am at high risk for diabetes would upset me” [italics added here for emphasis] to “Learning that I am at high risk for debt would upset me” [italics added here for emphasis]. Participants then showed their degree of agreement with each statement on a 7-point Likert scale ranging from 1 (strongly disagree) to 7 (strongly agree). After this, participants completed a debt-risk calculator in which their responses would be used “to calculate your chances of having debt problems in the next 5 years.” Participants completed 12 questions on topics such as gender, severity of debt and declarations of bankruptcy. They were then told their responses were used to calculate their future risk of debt, and that they had the choice to view this risk if they wished. We chose this this task and dependent measure for two reasons. Firstly, it ensured a degree of consistency as we modified a task (i.e., “diabetes risk calculator” to “debt-risk calculator”) and dependent measure (i.e., “choice to view risk of diabetes” to “choice to view risk of debt”) that had been successfully employed by Howell and Shepperd (2013). Secondly, the evidence suggests that between 75 and 83% of people will avoid dealing with the debt(s) (see Hatcher, 1994; Money Advice Service, 2013). Thus, we used a financial stimulus which was analogous to what people tend to avoid in the “real world.” In other words, this task mimicked a threatening debt-related situation, which allowed us to quantify the effect of contemplation on avoidance (or not) of debt-related information. Participants in the control condition were not exposed to any intervention prior to the dependent variable.

## Results of study 1

**Figure 1:**
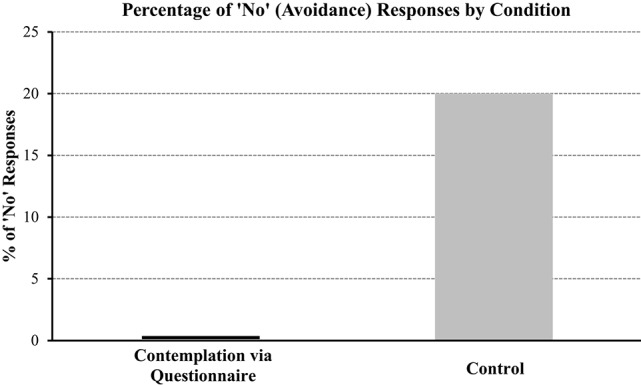
**Percentage (%) of “No” (avoidance) responses by condition (contemplation via questionnaire vs. control)**.

A Chi-square test of independence was used to compare the frequency of avoidance responses for participants in the contemplation to the control condition. A significant effect was revealed (χ^2^, (1, *N* = 40) = 4.44, *p* < 0.05). As represented in Figure 1, we can see that participants in the contemplation condition were numerically and statistically less likely to avoid viewing their risk of debt compared to those in the control condition (0% vs. 20% avoidance, respectively).

## Study 2: using a video to prompt contemplation of debt-related avoidance

In Study 1 we saw that prompting contemplation *via questionnaire* was effective in reducing the avoidance and debt-related information, now in Study 2 we test a similar proposition but this time prompt contemplation *via video*.

### Participants

*N* = 342 participants who volunteered through a large university in the UK “volunteers list” participated in this study. The average age of participants was 27.60 (*SD* = 11.04) with 301 females and 41 males. Participants were offered the chance of winning a £50 Amazon Voucher for taking part. British Psychological Society ethical requirements were met, including providing informed consent, and the option of debriefing when the study was completed by emailing the lead experimenter.

### Apparatus, design, and procedure

**Image 1:**
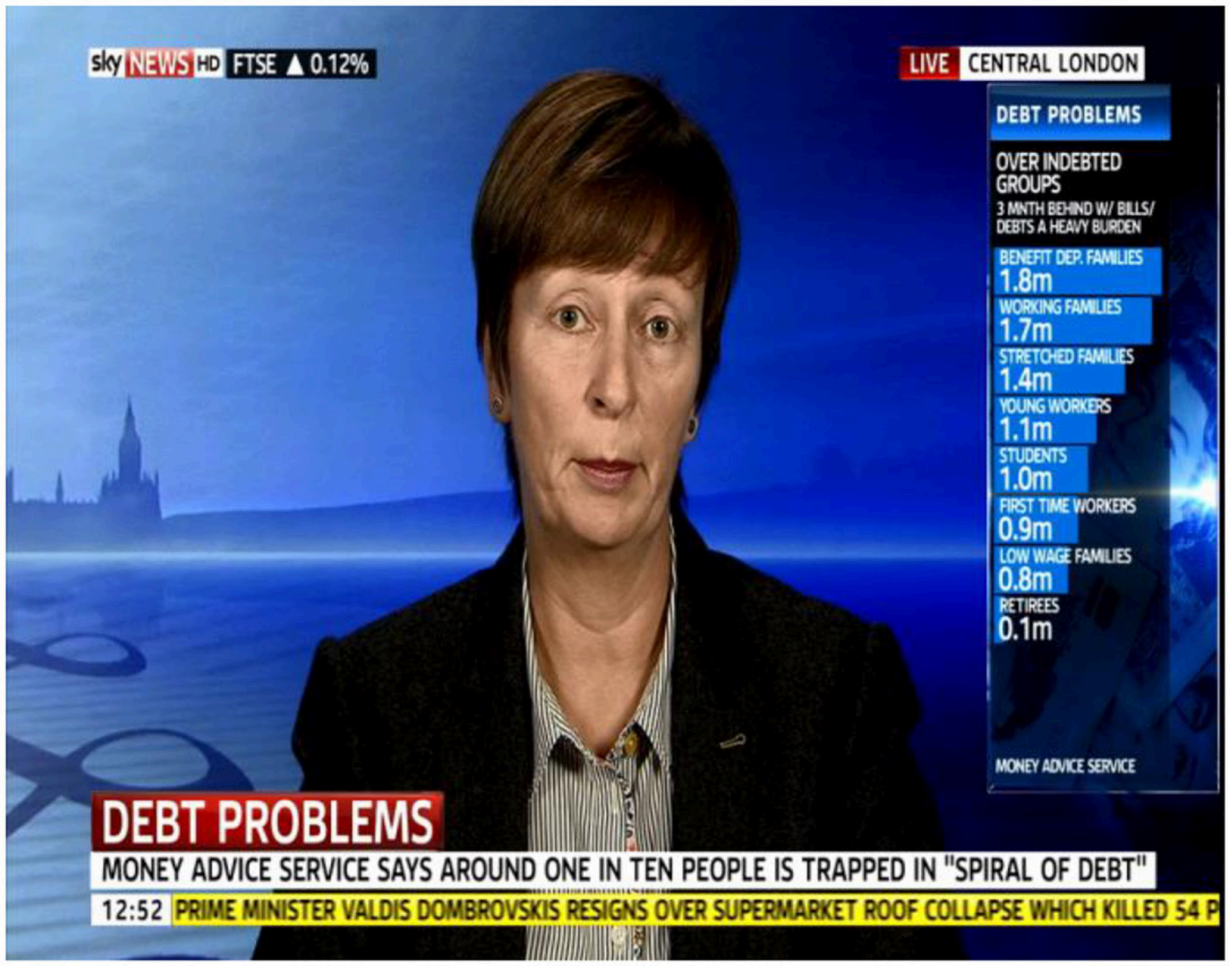
**Snapshot from Gullian Guy interview on Sky News**.

Qualtrics software (Qualtrics, Provo, UT) was used to collect the data. This study employed the same design, randomization procedure, and dependent measure of Study 1. The primary methodological difference between Studies 1 and 2 was that the former prompted contemplation via questionnaire where we now used a video. We now asked participants to watch a short (32 s) video and consider how the contents of the video applied to them. In this video chief executive of the Citizens Advice, Gullian Guy (see Image 1) discussed why avoiding debt makes it worse (i.e., prompting contemplation the cons of avoiding debt) and what solutions/advice are available (i.e., prompting contemplation of the pros of getting help). Specifically, she identified and provided solutions to the three components (i.e., threatens beliefs, negative emotions, undesired behaviors) that Howell and Shepperd (2013) identified as necessary for motivating avoidant behavior: (i) Debt denial/avoidance/shame: avoiding debt makes the problem worse and that being ashamed may contribute to this avoidance, (ii) Debt advice awareness/access: people do not know what debt advice is available or how to access it, and (iii) Timely intervention works best: the earlier people get debt advice the less likely they are to get into even more serious debt. As a result, this video remains analogous to the previous contemplation research as it prompts participants to consider the cognitive, emotional, and behavioral outcomes of avoidance generally (i.e., Howell and Shepperd, 2013) and debt specifically (i.e., Study 1 above). After participants viewed the video they completed the same dependent measure used in Study 1. Participants in the control condition were not exposed to any intervention prior to the dependent variable.

## Results and discussion of study 2

**Figure 2:**
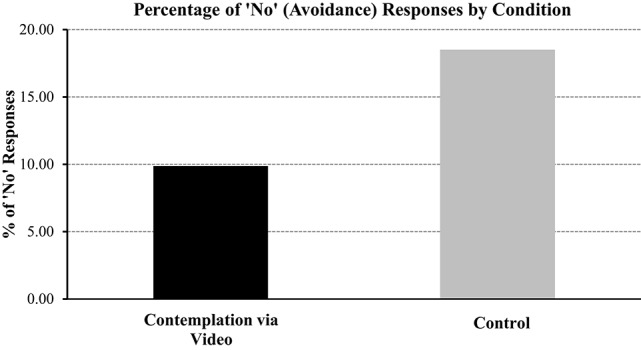
**Percentage (%) of “No” (avoidance) responses by Condition (contemplation via video vs. control)**.

A Chi-square test of independence was used to compare the frequency of avoidance responses for participants in the contemplation to the control condition. A significant effect was revealed (χ^2^, (1, *N* = 342) = 5.14, *p* < 0.05). Conform to our hypothesis and as we can see in Figure 2, prompting participants to contemplate the cons of avoiding and pros of not avoiding debt-related information *via video* reduced their avoidance of debt-related information, compared to those in the control condition (9.80% vs. 18.52% avoidance, respectively; see Figure 2). Not only did this highlight that contemplation is effective when prompted via video it supported the finding from Study 1: Contemplation is a generally effective intervention that reduces avoidance of debt-related information.

## Study 3: improving estimates of expenditure in an applied setting

In Studies 1 and 2 we saw that prompting contemplation (via questionnaire and video) was effective in reducing avoidance of debt-related information. We now focus on an area where avoidance likely has a negative effect on financial outcomes, i.e., underestimating expenditure (see Shapiro and Burchell, 2012; Santander, 2016). Therefore, in Study 3 we investigated if prompting participants to contemplate their expenditure will improve the quality of the estimates of expenditure that they provide.

### Participants

All participants were applying for a loan at credit union. Of the original 200 loan applications that were given out, 99 were returned. Eighteen were rejected as they failed to provide data from which subsequent analyses could be conducted. This left *N* = 81 suitable for statistical analysis. The demographic information of these 81 participants is provided in Table 1, and covers areas such as age, gender, number of children, housing type, any outstanding county court judgments, if they have ever declared bankruptcy, used high cost lenders, etc. British Psychological Society ethical requirements were met, including providing informed consent, and the option of debriefing when the study was completed by emailing the lead experimenter.

### Materials and procedure

#### Loan application booklet

Participants who came in to the credit union office and requested a loan were given a loan application booklet. On page one, applicants were asked to complete the form in its entirety; include all requested documentation; give evidence of all income/benefits; and show if they have any outstanding county court judgments, ever declared bankruptcy, used high cost lenders, and/or used a social fund loan. Page two asks for personal and demographic information, and the amount and purpose of the loan. Finally, on page three the applicant completes an expenditure (31 options, e.g., mortgage/rent) and income (8 options, e.g., wages) column. The column next to this was for “Office Use,” staff used this to verify with the applicants estimates of expenditure and income.

#### The intervention

Participants in the experimental condition completed six contemplation items which focussed on expenditure habits followed by the expenditure and income table discussed above. Instructions were given to read each question and consider how each applied to them. Those in the control condition only completed an estimate of their expenditure and income. To begin with we modified the 18 items from the original Howell and Shepperd (2013) study to apply to habits of expenditure. However, after discussion with the manager at the credit union they were further simplified in terms of number (18–6 items) and response measure (7-point Likert scale to a “yes” or “no” response). The staff suggested that these amendments were necessary to make sure that their clients understood and completed all the questions, thus keeping attrition to a minimum. Despite these changes great effort went into maintaining the core themes of the original questionnaire while applying it to expenditure, e.g., “It would be useful to know my risk for diabetes” was modified to “Knowing how I spend my money would be useful to me.”

#### Design

Half of the participants were randomly given a loan application form in its original form (control condition); the other half received the form with the contemplation items placed within it (experimental condition). British Psychological Society ethical requirements were met.

### Results

The three main dependent measures were: (i) *Thoroughness*: Number of items completed for expenditure and income; (ii) *Total*: Aggregate of expenditure and income estimates; and (iii) *Discrepancy*: Difference in the above expenditure measures between the applicants and the staff.

#### Applicants underestimate their expenditure

A paired samples *t*-test was used to compare total expenditure for what clients provided compared to when staff calculated it. This served as a reality check, and showed that clients in the control condition underestimated their total expenditure compared to when staff calculated it [550.32 vs. 690.28; *t*_(36)_ = 3.19, *p* < 0.01]. There was no comparable statistical difference in income estimates [812.85 vs. 812.72; *t*_(44)_ = 0.00, *p* > 0.05]. Applicants provided significantly less expenditure information compared to staff [10.68 vs. 14.43; *t*_(36)_ = 6.78, *p* < 0.01], with no comparable difference for number of income items [3.16 vs. 3.30; *t*_(36)_ = 1.30, *p* > 0.05]. Underestimating their total expenditure and using an incomplete number of expenditure items justifies the use of an intervention which targets improving expenditure estimates in this group.

#### Contemplation improves thoroughness

**Figure 3:**
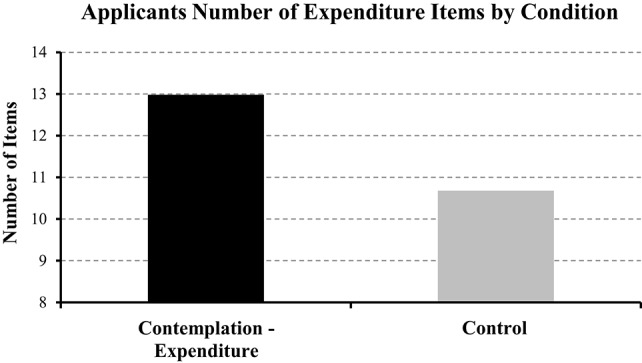
**Number of expenditure items provided by clients in the contemplation vs. control condition**.

Conform to expectations an independent-samples *t*-test revealed that applicants provided significantly more expenditure information in the experimental compared to the control condition [12.98 vs. 10.68; *t*_(79)_ = 2.84, *p* < 0.01; Figure 3], a pattern not observed when staff completed the form [15.25 vs. 14.43; *t*_(79)_ = 1.24, *p* > 0.05]. In contrast, there was no significant difference in number of income items between experimental and control conditions for applicants [*t*_(79)_ = 0.99, *p* > 0.05] or staff [*t*_(79)_ = 0.82, *p* > 0.01].

#### Contemplation improves expenditure totals

**Figure 4:**
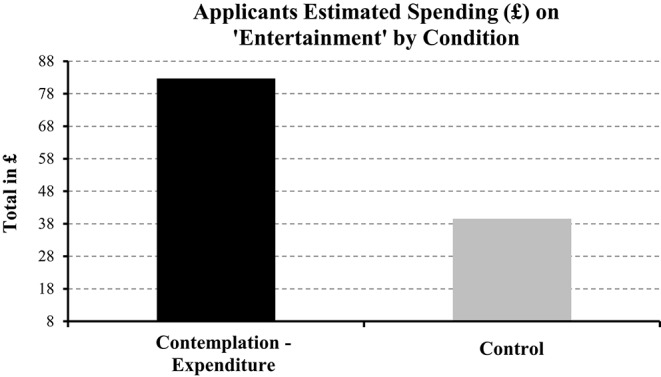
**Estimated spending (£) on “entertainment” by clients in the contemplation vs. control condition**.

An independent-samples *t*-test revealed that for total expenditure and income estimates there were no significant differences for applicants or staff in the experimental vs. control comparisons (all *p* > 0.05). However, and as depicted in Figure 4, for a group of expenditure items defined as “entertainment” (e.g., birthday presents, Christmas), applicants provided larger estimates when in the experimental compared to the control condition [82.65 vs. 39.52; *t*_(79)_ = 2.74 *p* < 0.05; Figure 4], a similar pattern was observed in the estimates of staff [119.15 vs. 78.61; *t*_(79)_ = 2.04, *p* < 0.05].

#### Contemplation improves agreement between applicants and staff

**Figure 5:**
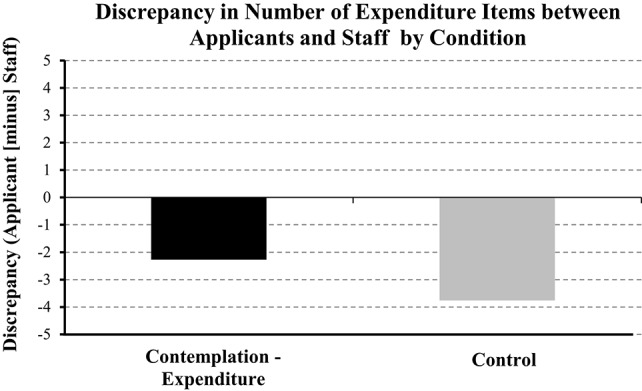
**Discrepancy between number of expenditure items between applicants and staff**. A value closer to zero indicates greater agreement between applicants and staff.

We then compared the discrepancy between applicants and staff for the above measures. An independent-samples *t*-test revealed was a significant difference *t*_(79)_ = 2.34, *p* < 0.05 in the discrepancy (staff [minus] applicants) for the number of expenditure items they provided between the experimental and control condition. As shown in Figure 5 a lower discrepancy value of −2.27 in the experimental compared to the control condition (−3.76), indicates more agreement between staff and clients in the former compared to the later condition. This provides more evidence that the present intervention was effective. In that, as staff verify each expenditure item this increases the total number of expenditure items identified. Participants in the contemplation condition produced a number of expenditure items which agreed with those identified by staff to a greater extent than those in the control condition. In contrast, there was no significant difference in the discrepancy for number of income items provided [*t*_(79)_ = 0.36, *p* > 0.05]. For total expenditure estimates discrepancy was less for those in the experimental (−76.92) compared to the control condition (−139.96), however, this numerical difference only produced a weak trend toward significance [*t*_(79)_ = 1.38, *p* = 0.17]. In contrast, there was no distinct numerical or statistical difference in total income estimates between conditions [0.05 vs. 0.14; *t*_(79)_ = −0.00, *p* > 0.05].

## Discussion of study 3

Loan applicants underestimated their total expenditure by ≤ 150 per month, which justified the use of an intervention designed to improve this financial behavior. We observed that contemplation increased the number of expenditure items and total estimates of expenditure for “entertainment” that participants provided. This indicated that our intervention improved estimates of expenditure in specific areas. Secondly, contemplation reduced the discrepancy between staff and applicants for the number of items included in the expenditure list. In that, once applicants handed in their form, staff verified each item of expenditure and asked why they not included an obvious item, e.g., birthday presents for children was commonly forgotten about. In other words, contemplation reduced the likelihood that an item of expenditure would be left off the list. In sum, these findings supported our applied hypothesis: Prompting loan applicants to contemplate their expenditure improved the estimates of expenditure that they provided.

## General discussion

The present research tackled two main areas of financial mismanagement, i.e., avoiding debt and underestimating expenditure. Thankfully our findings showed that contemplation reduced avoidance of debt-related information and improved the estimate of expenditure that participants provided. We found evidence for reduced avoidance in Studies 1 and 2, i.e., prompting participants to contemplate reasons for avoiding debt-related information were less likely to avoid viewing debt-related information. Importantly, we observed this effect when contemplation occurred via questionnaire (Study 1) and video (Study 2). Regarding our applied hypothesis, in Study 3 we observed that prompting loan applicants to contemplate their expenditure improved the estimates of expenditure that they provided. Those in the contemplation condition wrote down more expenditure items and provided larger (i.e., closer to real expenditure) estimates for “entertainment” (e.g., going out, birthday presents, Christmas, hobbies) compared to those in the control condition. Finally, contemplation resulted in greater agreement between applicants and staff in terms of the number of expenditure items that applicants wrote down. We conclude that contemplation is a robust intervention as it was effective for separate research groups investigating different areas of avoidance (debt; the present research vs. health; Howell and Shepperd, 2013); and for different types of interventions (questionnaire; Study 1 vs. video; Study 2), behaviors (avoidance of debt-related information; Studies 1 and 2 vs. quality of expenditure information; Study 3), and samples (students and university staff; Studies 1 and 2 vs. loan applicants at a credit union; Study 3).

### Impact of the present findings

The extant literature invariably reports that people are poor at managing their finances (e.g., National Savings and Investment Survey, 2012; Santander, 2016), and identifies a plethora of psychological and sociodemographic factors which underlie it (e.g., Livingstone and Lunt, 1992; Lea et al., 1993; Webley and Nyhus, 2001; Brown et al., 2005; Bernerth et al., 2012). In contrast, there is much smaller body of research (i.e., Ulkuemen and Cheema, 2011; Bryan and Hershfield, 2012; Tam and Dholakia, 2014) showing that improvements in financial decision-making can occur via simple interventions. A point supported by the finance researcher Winerman (2004): “At present … I don't think we have the research base to know exactly what is needed” to reduce problem debt (p. 62). As such we propose that the present findings make the following four-fold contribution to the literature.

(1). *Contemplation is effective in a new domain*. Howell and Shepperd (2013) questioned the effectiveness of contemplation in domains other than health. Fortuitously, we showed that two key processes involved in financial mismanagement (namely, avoidance of debt-related information and underestimation of expenditure) improved as the result of contemplation. This suggests that contemplation is likely useful in domains where avoidance impairs task performance, e.g., few people know how much household energy they use, or food/alcohol they have consumed (Polivy, 1976; Hull, 1981; Abrahamse et al., 2005).

(2) *Contemplation via video reduces avoidance*. Prompting contemplation via video decreased the likelihood that people would then avoid looking at their risk of debt. This suggests that TV campaigns which encourage people to get help for their financial problems will benefit from including aspects of contemplation. The need for such empirically based TV adverts has never been more in demand, as the number of adverts which encourage financial mismanagement has exploded since their deregulation in 2007. As a result since then to 2012 there has been a 3,500% and 600% increase in the number of high interest pay-day loan and gambling adverts on our TVs, which equates to an adult viewing them ~152 and ~630 times in a year, respectively (see Ofcom, 2013a,b). If we agree that the primary aim of these adverts is to encourage people to use pay-day loans and/or gamble, and that such financial behaviors are associated with poorer financial and social outcomes (see Hong et al., 2009), then any intervention which can decrease and/or temper their use is both necessary and welcome.

(3) *Contemplation improves estimates of expenditure*. We observed that contemplation improved the estimates of expenditure that people provided. As such we satisfied what DeVaney and Lytton (1995) stated was the need for people to: “Recognize the fundamental need for planned spending and managed cash flow [, i.e., the] calculation and tracking of net worth, income, and expenses” (p. 153). In effect, the present intervention likely encouraged people to think and act about their finances in a way they may not otherwise have done. This suggestion is consistent with a finding from a meta-analysis which examined the effectiveness of different meta-cognitive strategies on academic performance (i.e., math, sciences, reading, and writing; Donker et al., 2014). Specifically, they reported that encouraging people to think more deeply (i.e., elaboration) about a math problem improved their ability to solve the problem. This suggests that financial management may have benefited from people using similar meta-cognitive strategies to those which benefited math performance, strategies which encourage people to go beyond the superficial and perhaps avoidant processes that they would normally use.

(4) *Contemplation is effective within an applied setting*. For the first time we showed that a simple intervention could improve financial decision-making in an applied setting. This finding has added importance as loan applicants have a range of negative experiences with financial institutions (e.g., prevalent use of high cost lenders; see Table 1), which likely contributes to further avoidance of financial matters and the strengthening of a refusal to talk about finances with others (i.e., see taboo of discussing finances; Atwood, 2012). Contemplation provided a non-invasive and non-embarrassing means of encouraging this group to think and possibly talk about their expenditure in a way that they would not normally have done. Thus, in a manner consistent with Prochaska and DiClemente's (2005) transtheoretical model of behavior change, contemplation may have reduced the influence of negative financial expectancies/experiences (e.g., fear of knowing that finances are in a bad situation/embarrassment; see Yakoboski and Dickemper, 1997 for evidence of this), and increased the likelihood of positive financial behaviors (e.g., thinking about/looking at expenditure in more detail; Yeung and Summerfield, 2012). As such contemplation may prove useful for similarly complex groups with entrenched and socially embarrassing behaviors. For example, employment agencies could use contemplation (e.g., highlight the pros of seeking employment: money, self-esteem, social mobility vs. the cons of not seeking employment: no money, less self-esteem, less social mobility) to complement present methods of getting people back into employment (see Noordzij et al., 2013).

### Limitations and future research

We identify the following limitations to the present research, and propose areas of future research where appropriate. Firstly, while we proposed that contemplation reduced avoidance and improved financial estimations, the exact mechanism which mediates this has not been identified. For example, the transtheoretical model of behavior change would predict that contemplation would result in the pros of change outweighing the cons of changing, which in turn would mediate subsequent reductions in avoidance (see Prochaska et al., 2008). It is advisable for future research to tease apart the mediating effect of these as well as other measurable psychological factors of avoidance. For example, does contemplation result in quantifiable changes in factors identified as key to avoidance (i.e., beliefs and/or emotions, and/or actions; see Sweeny et al., 2010), and do these mediate the relationship between our interventions and dependent measures? Secondly, we are unsure if contemplation encouraged participants to engage in financial behaviors that they then used to improve their estimates of expenditure? In that, were they more likely to check their bank balances online, use written calculations, and/or ask a partner/child how much they spend in a given area? Future research could investigate if contemplation changes the frequency and nature of financial monitoring, and if such changes result in more accurate financial estimates. For example, in a review conducted by Harkin et al. (2016) one of their key findings was that changes in the frequency of progress monitoring mediated the effect of interventions on goal attainment. Therefore, a question for future research is whether changes in the frequency of financial monitoring mediated the effect of contemplation on improved estimates of expenditure? Thirdly, in Study 3 we focused on clients at a credit union, which poses the question: Would these findings would generalize to those from different economic and sociodemographic backgrounds. In that, while university students are known to fall into debt, little (if any) research has provided interventions to improve this (see Norvilitis et al., 2006). Thus, investigating student's ability to make exact financial estimates and providing interventions similar to those employed here may help increase our understanding of this problem. Fourthly, a line of inquiry that our research did not address was to the long-term effect of our interventions, i.e., after the study finished did participants continues to show reduced avoidance of debt-related information and/or improved expenditure estimates? Lastly, the present paper did not examine the role of biases such as overconfidence, optimism, and the misuse of information in explaining financial mismanagement (see Sartori and Ceschi, 2013). For example, was it the case that contemplation resulted in improved financial optimism which then reduced avoidance of debt-related information and/or improved estimates of expenditure? Future research would be best served to examine if such factors mediate the relationship between contemplation and measures of financial management.

## Summary

People who avoid information either refuse to learn or avoid exposure to that information (see McCloud et al., 2013). Such information normally threatens an individual personally (i.e., beliefs and/or emotions) and/or requires a behavior that they do not want to carry out (see Sweeny et al., 2010). In certain instance debt and financial information satisfies both these criteria: People have a tendency to avoid dealing with their debt/finances as it likely threatens beliefs and/or emotions and/or requires undesired behaviors. Fortunately, Howell and Shepperd (2013) showed that prompting participants to contemplate the consequences of avoidance reduced avoidance of threatening information. This motivated us to use contemplation in the context of debt-related information and estimates of expenditure. Our findings showed that prompting participants to contemplate the consequences of avoiding debt-related information (Studies 1 and 2) and habits of expenditure (Study 3) reduced avoidance of debt-related information and improved estimates of expenditure, respectively. The robustness and flexibility of contemplation was further underlined by its success in an applied setting with a sample who had a complex financial history (Study 3). In sum, the present findings contribute to the financial literature in the following ways: (1) asking people to contemplate reasons for avoiding debt-related information can reduce the likelihood that they will then avoid it, (2) that contemplation can successfully be induced via questionnaire and video, (3) that the common problem of underestimating expenditure can be improved using a simple contemplation intervention, and (4) that financial mismanagement can be improved in an applied setting for groups with complex and negative financial histories.

## Ethics statement

This study was carried out in accordance with the recommendations of the ethics committee at the Department of Psychology, University of Sheffield with written informed consent from all subjects. All subjects gave written informed consent in accordance with the Declaration of Helsinki. The protocol was approved by the ethics committee at the Department of Psychology, University of Sheffield.

## Author contributions

BH conducted all of the design, data collection, data-analysis, and write-up of all the three studies.

## Funding

BH is funded by Economic and Social Research Council (ESRC) Future Leaders Research Grant (ES/K008986/1). The funding body had no involvement in design, data collection, analysis or write-up of this paper.

### Conflict of interest statement

The author declares that the research was conducted in the absence of any commercial or financial relationships that could be construed as a potential conflict of interest.
